# Supplementary material for: Non-invasive red-light optogenetic control of Drosophila cardiac function
Source: Commun Biol. 2020 Jun 29;3:336. doi: 10.1038/s42003-020-1065-3 (PMC7324573; doi:10.1038/s42003-020-1065-3)
Supplement: Supplementary file 1 — Supplementary Information [file 42003_2020_1065_MOESM1_ESM.pdf]

Supplementary Figures

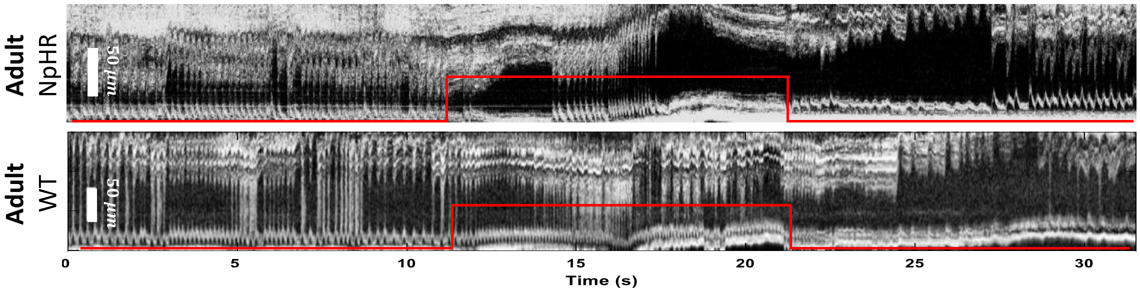

Supplementary Figure 1. M-mode images demonstrating inhibition of cardiac function with 10 s constant red-light stimulation of NpHR and WT adult flies. Short term inhibition of cardiac function was observed in NpHR flies after the red light was turned on, while no response to red light stimulation was observed within WT flies.

Supplementary Tables

Supplementary Table 1. Characterization of fruit fly lines for red-light optogenetic pacing, listing the fly stock and number of screened flies for *UAS-ReaChR; 24B-GAL4* and *20×UAS-IVS-CsChrimson; 24B-GAL4* flies respectively. All flies were cultured at 25 ℃. No: unable to be paced; Yes: able to be paced.

**Opsin**	***UAS-ReaChR; 24B-GAL4***							***20×UAS-IVS-CsChrimson; 24B-GAL4***		
**Fly Stock #**	53740	53741	53742	53746	53747	**53748**	53749	55134	55135	55136
**Number of flies (n)**	7	6	4	3	4	**38**	10	21	48	49
**Paceability**	No	No	No	No	No	**Yes**	Yes	Yes	Yes	Yes
**Note**	-	-	-	-	-	**Good pace-ability**	Eclosion failure	Only females were paceable	Eclosion failure	Eclosion failure

Supplementary Table 2. The number of *UAS-ReaChR; 24B-GAL4* flies measured at different developmental stages for characterization of pacing pulse width, power density, and pacing frequencies.

**Parameters/developmental stages**	**Larva**	**Early pupa**	**Late pupa**	**Adult**
**Optimize pulse width (n)**	14	17	11	12
**Optimize power density (n)**	14	17	11	12
**Characterize pacing frequency (n)**	14	16	11	10

Supplementary Table 3. The number of *UAS-NpHR-YFP; 24B-GAL4* flies measured in the larval, early pupal, and late pupal developmental stages for determining the needed excitation power density to induce cardiac arrest.

**Fly model/ developmental stages**	**Larva**	**Early pupa**	**Late pupa**
UAS-NpHR-YFP; 24B-GAL4 **(n)**	10	10	9

Supplementary Notes

I. Fly Line Development and Assessment

A. Characterization of fruit fly lines for red-light excitatory pacing

We developed fly models for red light excitatory pacing by crossing *24B-GAL4* with *UAS-ReaChR* and *UAS-CsChrimson* flies obtained from Bloomington Drosophila Stock Center, including fly lines #53740, #53741, #53742, #53746, #53747, #53748, #53749, #55134, #55135, and #55136 (Supplementary Table 1). The red-shifted microbial opsins ReaChR and CsChrimson were targeted for cardiac tissue expression through *UAS* mediation and the *24B-GAL4* driver system. ReaChR and CsChrimson flies were mated and cultured on Formula 4-24 (Instant *Drosophila* Medium; Carolina Biological Supply Company) with all-*trans*-retinal (ATR) (Toronto Research Chemicals Inc.). The first generation of each fly stock was cultured at 25 ℃ and tested by red light optogenetic pacing in the larval, early pupal, and adult stages. M-mode images obtained with the OCM system showed no change in heart function for the #53740, #53741, #53742, #53746, and #53747 flies, indicating unsuccessful pacing. CsChrimson #55134 flies could be paced in the larval and pupal stages, but only female flies could be successfully paced. Pacing was successfully performed in the larval and pupal stages of the #53749, #55135, and #55136 stocks, but the flies were not able to undergo eclosion. A heartbeat pause was also observed in #53749 larval flies after red light stimulation. The heart restarted beating after ~5 minutes. The first generation of the ReaChR #53748 fly stock were able to develop into adults and were able to be paced throughout their lifecycle. This optimized fly stock was used to demonstrate excitatory pacing and simulate tachycardia.

B. Characterization of fruit fly lines for simulating cardiac arrest

The *UAS-NpHR-YFP; 24B-GAL4* transgenic fruit fly models were engineered by crossing the *UAS-NpHR-YFP* fly from Bloomington Drosophila Stock Center (#41752) with *24B-GAL4* flies.

The same culturing protocol as previously described was used, but with an ATR concentration of 10 mM to ensure sufficient expression of NpHR in cardiomyocytes. The first generation flies were evaluated at the larval stage by stimulating the heart for 4 s at a power density of 7.26 mW/mm^2. Heart beat inactivation was observed in ten flies in response to the red-light illumination. The *UAS-NpHR-YFP; 24B-GAL4* flies were then used for mimicking cardiac arrest and bradycardia.

Constant red-light stimulation was applied to the larva, early pupa, late pupa, and adult NpHR flies. Inhibition of cardiac function was observed in the first three developmental stages. In *UAS-NpHR-YFP; 24B-GAL4* adult flies, red light stimulation could cause the heart to pause for 1 to 2 seconds (Supplementary Figure 1a), but WT adults had no response to the red-light stimulation (Supplementary Figure 1b). The fly heart undergoes remodeling during metamorphosis, which might affect the NpHR expression level, leading to partial failure of cardiac inhibition in adult flies. The high metabolic level of adult fly heart may also prohibit it from stopping for over a few seconds with red light inhibition.

II. ReaChR flies used for characterization of red-light pulses for excitatory pacing

To characterize excitation pulses for successfully pacing ReaChR flies, the excitation power density, pulse width, and pacing frequency were investigated at each developmental stage. ReaChR larva, early pupa, late pupa, and adult flies were paced and imaged using our integrated red LED and OCM system. The flies studied are listed in Supplementary Table 2.

III. NpHR flies used for characterization of the excitation power density needed to mimic cardiac arrest

The correlation of the cardiac arrest probability with excitation power density was determined by tuning the red-light power from 0.12 mW/mm^2 to 7.26 mW/mm^2. A cardiac arrest lasting 10 s was tested in multiple flies in the larval, early pupal, and late pupal stages (Supplementary Table 3). Excitation light power densities of 3.63 mW/mm^2, 0.46 mW/mm^2, and 7.26 mW/mm^2, respectively were optimal for inducing continuous cardiac inhibition for 10 s.
