# Supplementary material for: Non-invasive red-light optogenetic control of Drosophila cardiac function
Source: Commun Biol. 2020 Jun 29;3:336. doi: 10.1038/s42003-020-1065-3 (PMC7324573; doi:10.1038/s42003-020-1065-3)
Supplement: Supplementary file 2 — Description of Additional Supplementary Files [file 42003_2020_1065_MOESM2_ESM.pdf]

Description of Additional Supplementary Files

Supplementary Data 1:
Source data used in generating Figure 3b, d, e-h (Characterizations of optogenetic excitatory pacing of individual fruit flies), Figure 4c (Characterizations of optogenetic inhibitory pacing of individual fruit flies), and Figure 5c-d (Characterizations of cardiac recovery after optogenetic cardiac arrest in individual fruit flies).

Supplementary movie 1 –Red-light excitatory pacing of a ReaChR larval fly. The heart of the larval fly beat at the resting heart rate (RHR) of 2.8 Hz initially, then followed the stimulation pulses at a frequency of 4.5 Hz, and returned to the RHR after the red-light stimulation was suspended.

Supplementary movie 2 –Red-light excitatory pacing of a ReaChR early pupal fly. The heart of the early pupa beat at the RHR of 1.4 Hz initially, then followed the stimulation pulses at a frequency of 3 Hz, and returned to the RHR after the red-light stimulation was suspended.

Supplementary movie 3 - Red-light excitatory pacing of a ReaChR late pupal fly. The heart of the late pupa beat at the RHR of 2.5 Hz initially, then followed the stimulation pulses at a frequency of 5 Hz, and returned to the RHR after the red-light stimulation was suspended.

Supplementary movie 4 - Red-light excitatory pacing of a ReaChR adult fly. The heart of the adult fly beat at the RHR of 8.1 Hz initially, then followed the stimulation pulses at a frequency of 10.5 Hz, and returned to the RHR after the red-light stimulation was suspended.

Supplementary movie 5 - Red-light stimulation induces cardiac arrest in an NpHR larval fly.

The heart of the larval fly beat at the RHR of 4.8 Hz for the first 10 s with no red-light stimulation. It suspended beating immediately after the red light was turned on, and remained in a relaxed state for the 10s duration of red-light illumination. The heart resumed beating after the red light was turned off.

Supplementary movie 6 - Red-light stimulation induces cardiac arrest in an NpHR early pupal fly. The heart of the early pupa beat at the RHR of 2.2 Hz for the first 10 s with no red-light stimulation. It suspended beating immediately after the heart was illuminated and remained in a relaxed state for the 10s duration of red-light illumination. The heart resumed beating after the red light was turned off.

Supplementary movie 7 - Red-light stimulation induces cardiac arrest in an NpHR late pupal fly. The heart of the late pupal fly beat at the RHR of 1.6 Hz for the first 10 s. It suspended beating immediately after the heart was illuminated and stayed in a relaxed state for the 10s duration of red-light illumination. The heart resumed beating after the red light was turned off.

Supplementary movie 8 - Red-light inhibitory pacing of an NpHR larval fly. The heart of the larval fly beat at the RHR of 3.4 Hz, slowed down to 1 Hz with a pulse frequency of 1 Hz and

pulse duty cycle of 90%, and the heart rate returned to the RHR after the red-light illumination was suspended.

Supplementary movie 9 - Red-light inhibitory pacing of an NpHR early pupal fly. The heart of an early pupa beat at the RHR of 2.7 Hz, slowed down to 1 Hz with a pacing frequency of 1 Hz and a pulse duty cycle of 80%, and the heart rate returned to the RHR after the red-light illumination was suspended.

Supplementary movie 10 - Red-light inhibitory pacing of an NpHR late pupal fly. The heart of the late pupal fly beat at the RHR of 3.3 Hz, slowed down to 1 Hz with a pulse frequency of 1 Hz and a pulse duty cycle of 70%, and the heart rate returned to the RHR after the red-light illumination was suspended.

Supplementary movie 11 – Cardiac recovery after red-light stimulation for 1 s in an NpHR early pupal fly. The fly heart rate quickly returned to the RHR after the red-light illumination was suspended.

Supplementary movie 12 – Cardiac recovery after red-light stimulation for 2 s in an NpHR early pupal fly. The fly heart rate quickly returned to the RHR after the red-light illumination was suspended.

Supplementary movie 13 – Cardiac recovery after red-light stimulation for 5 s in an NpHR early pupal fly. After the red-light illumination was suspended, an overshoot of HR was observed followed by a gradual HR recovery to the RHR.

Supplementary movie 14– Cardiac recovery after red-light stimulation for 10 s in an NpHR early pupal fly. After the red-light illumination was suspended, an overshoot of HR was observed followed by a gradual HR recovery to the RHR.

Supplementary movie 15 – Cardiac recovery after red-light stimulation for 20 s in an NpHR early pupal fly. After the red-light illumination was suspended, an overshoot of HR was observed followed by a gradual HR recovery to the RHR.
